# The relation between exercise and glaucoma in a South Korean population-based sample

**DOI:** 10.1371/journal.pone.0171441

**Published:** 2017-02-10

**Authors:** Shuai-Chun Lin, Sophia Y. Wang, Louis R. Pasquale, Kuldev Singh, Shan C. Lin

**Affiliations:** 1 Department of Ophthalmology; University of California San Francisco; San Francisco, California, United States of America; 2 Department of Medicine, National Yang-Ming University, Taipei, Taiwan; 3 Kellogg Eye Institute, University of Michigan, Ann Arbor, Michigan, United States of America; 4 Massachusetts Eye and Ear Infirmary, Glaucoma Service, Harvard Medical School, Boston, Massachusetts, United States of America; 5 Channing Division of Network Medicine, Brigham and Women’s Hospital, Harvard Medical School, Boston, Massachusetts, United States of America; 6 Department of Ophthalmology; Stanford University; Stanford, California, United States of America; Bascom Palmer Eye Institute, UNITED STATES

## Abstract

**Purpose:**

To investigate the association between exercise and glaucoma in a South Korean population-based sample.

**Design:**

Population-based, cross-sectional study.

**Participants:**

A total of 11,246 subjects, 40 years and older who underwent health care assessment as part of the 2008–2011 Korean National Health and Nutrition Examination Survey.

**Methods:**

Variables regarding the duration (total minutes per week), frequency (days per week), and intensity of exercise (vigorous, moderate exercise and walking) as well as glaucoma prevalence were ascertained for 11,246 survey participants. Demographic, comorbidity, and health-related behavior information was obtained via interview. Multivariable logistic regression analyses were performed to determine the association between the exercise-related parameters and odds of a glaucoma diagnosis.

**Main outcome measure(s):**

Glaucoma defined by International Society for Geographical and Epidemiological Ophthalmology criteria.

**Results:**

Overall, 336 (2.7%) subjects met diagnostic criteria for glaucomatous disease. After adjustment for potential confounding variables, subjects engaged in vigorous exercise 7 days per week had higher odds of having glaucoma compared with those exercising 3 days per week (Odds Ratio [OR] 3.33, 95% confidence interval [CI] 1.16–9.54). High intensity of exercise, as categorized by the guidelines of the American College of Sports Medicine (ACSM), was also associated with greater glaucoma prevalence compared with moderate intensity of exercise (OR 1.55, 95% CI 1.03–2.33). There was no association between other exercise parameters including frequency of moderate exercise, walking, muscle strength exercise, flexibility training, or total minutes of exercise per week, and the prevalence of glaucoma. In sub-analyses stratifying by gender, the association between frequency of vigorous exercise 7 days per week and glaucoma diagnosis remained significant in men (OR 6.05, 95% CI 1.67–21.94) but not in women (OR 0.96 95% CI: 0.23–3.97). A U-shaped association between exercise intensity and glaucoma prevalence was noted in men (OR 1.71, 95% CI 1.09–2.69 for low intensity versus moderate intensity; OR 2.19, 95% CI 1.25–3.85 for high intensity versus moderate intensity).

**Conclusion:**

In a South Korean population sample, daily vigorous exercise was associated with higher glaucoma prevalence. In addition, the intensity of exercise was positively associated with glaucoma diagnosis in men but not women.

## Introduction

Regular physical activity is a healthy lifestyle with numerous benefits to general health including the reduced risk of common ophthalmic diseases.[1, 2] An animal study demonstrated that daily swimming in middle-aged mice protected retinal ganglion cells against age-related functional loss and signs of stress after an acute injury.[3] Evidence has shown that intraocular pressure (IOP) is transiently reduced by dynamic and isometric exercise while exercise also increases systolic blood pressure through sympathetic stimulation and results in an increase in ocular perfusion pressure (OPP).[4–6] Although the mechanism is not completely understood, a constant and adequate ocular blood flow is maintained by autoregulation during these perfusion pressure changes.[7–9] However, dysfunctional autoregulation of ocular blood flow during exercise has been noted in diabetic autonomic neuropathy patients.[10, 11]

The effects of exercise on glaucoma risk are not completely clear. Some physical activities such as weightlifting and selected yoga postures can cause transient IOP elevation by increasing intra-thoracic pressure, which may cause glaucomatous damage in vulnerable subjects.[12, 13] Patients with pigment dispersion syndrome also experience elevated IOP and transient visual disturbance after exercise.[14] Furthermore, the beneficial effects of exercise may be lost with exhaustion. It is well known that exhaustive exercise causes structural damage or inflammatory reactions within the muscles due to the production of free radicals[15], and evidence indicates that oxidative stress plays a major role in glaucoma.[16–18]

Williams et al.[19] demonstrated decreases in glaucoma incidence with each kilometer (km) per day run and with each per meter per second increment in speed in a 10-km footrace performance among 29,854 male runners followed for 7.7 years. However, the diagnosis of glaucoma was based on self-report, and the authors did not evaluate other parameters such as the frequency or duration of running, or compare different exercise intensities. The precise biological mechanisms by which physical activity influences disease processes are complicated, and several parameters such as age, intensity, duration, and frequency of exercise could play a role.

Unlike prior work on diabetic retinopathy[20, 21], most major epidemiological studies investigating risk factors for glaucoma have not assessed the effects of physical activity on glaucoma prevalence. Therefore, we investigated the relationship between exercise patterns (intensity, duration, and frequency) and glaucoma prevalence in the Korean National Health and Nutrition Examination Survey (KNHANES), a large population-based survey conducted from 2008 through 2011, in South Korea.

## Methods

### Study population

All analyses were based on data from the fourth and fifth KNHANES performed from January 2008 to December 2011. The KNHANES is a cross-sectional survey that examines the health and nutritional status of the non-institutionalized civilian population of South Korea. The survey has been conducted annually since 2007 under the auspices of the Korea Centers for Disease Control and Prevention with approval by its institutional review board. The KNHANES consists of the health interview, health examination and nutrition surveys, and a health examination. The survey adheres to the principles outlined in the Declaration of Helsinki for research that involves humans, and all participants provide written informed consent. This nationwide representative study uses a stratified, multistage probability sampling design with a rolling survey sampling model.

Data on demographic characteristics, diet, and health-related variables were collected through personal interviews and self-administered questionnaires. Physical examination as well as blood and urine sampling were performed at a mobile examination center. Ophthalmologic interview questions and examinations were added in the second half of 2008 and are thus available for KNHANES IV and V. Response rates of 77.8% in 2008, 82.8% in 2009, 81.9% in 2010, and 80.4% in 2011 were obtained. Among 16,109 subjects age 40 years and older who participated during the 4-year study period and received an ophthalmologic examination, we excluded those who had a history of retinal disease or stroke to minimize the likelihood of non-glaucomatous visual field (VF) defects resulting in an erroneous diagnosis of glaucoma, and those without physical activity data, yielding 11,246 subjects for our analyses.

### Survey components

Data on demographic characteristics and health-related variables was collected through personal interview and a self-administered questionnaire. Physical examination and blood and urine collections were performed at a mobile examination center. Ophthalmologic interview questions and examinations were added in the second half of 2008 and thus were available for KNHANES IV. The ophthalmologic examinations conducted in the KNHANES have been described in prior publications.[22, 23] After an ophthalmology-focused interview, participants underwent visual acuity measurement, automated refraction, slit lamp examination, IOP measurement, fundus photography, and, when deemed appropriate, visual field (VF) examination with frequency doubling technology (FDT; Humphrey Matrix; Carl Zeiss Meditec, Inc, Dublin, CA). A digital nonmydriatic fundus camera (TRC-NW6S, Topcon, Tokyo, Japan) and a Nikon D-80 digital camera (Nikon, Tokyo, Japan) were used to obtain the digital fundus images. Images were captured from all participants 19 years and older under physiological mydriasis. For each participant, one 45° nonmydriatic digital retinal image centered on the fovea was taken per eye (2 images per person in total). Vertical cup-to-disc ratios (VCDR) were measured from digital fundus images.

The primary outcome variable was the presence or absence of a glaucoma diagnosis as defined by International Society of Geographical and Epidemiological Ophthalmology (ISGEO) criteria previously described.[24] Category 1 criteria are defined by a VF defect consistent with glaucoma and either vertical cup-to-disc ratio (VCDR) greater than or equal to 0.7 (97.5th percentile) or asymmetry of VCDR between right and left eyes greater than or equal to 0.2 (97.5th percentile). Category 2 criteria, utilized when VF results were not definitive or not available, required VCDR greater than or equal to 0.9 (99.5th percentile) or asymmetry of VCDR greater than or equal to 0.3 (99.5th percentile). Category 3 criteria, utilized when no information on VF testing or optic disc examination was available, required visual acuity less than 3/60 and IOP greater than the 99.5th percentile for this population (21 mmHg). Testing of visual function was performed with FDT in glaucoma suspects as defined by criteria described in previous studies[22, 23], and a defect was deemed present if two different test locations were abnormal. The test was repeated if the proportion of fixation errors or false positive responses were greater than 0.33. If a subject failed the fixation cutoff on two VF attempts, the test was considered invalid. Subjects who were unable to perform valid VF testing were then considered for a glaucoma diagnosis using ISGEO category 2 or 3 criteria.

### Measurements

#### Patterns of physical activity: Intensity, duration, and frequency

KNHANES IV and V consist of eight questions about physical activity, which measure physical activity patterns in the sample of the Korean population. These questions pertain to type of exercise (vigorous, moderate, walking, muscle strength training, and flexibility training); duration (how many hours and minutes per day); and frequency (how many days per week).[25, 26] Vigorous physical activities include running or jogging, hiking, fast bike riding, fast swimming, football, basketball, jumping rope, squash, singles tennis, heavy items lifting and athletic activities; moderate physical activities include swimming, doubles tennis, volleyball, badminton, table tennis, and lightweight lifting; and walking as mild physical activity.

Subjects were questioned about the level of physical activity in the following 3 categories based on the guidelines of the American College of Sports Medicine (ACSM)[27]: (a) Did you walk for at least 30 min, 5 times on a recent week? (b) Did you perform moderate physical activity for at least 30 min, 5 times on a recent week? and (c) Did you perform vigorous physical activity for at least 20 min, 3 times on a recent week? The physical activity levels were then further divided in 3 different intensities: (a) low, subjects who did not belong in any of 3 categories; (b) moderate, subjects who belong to 1 of the 3 categories; and (c) high, subjects who belong to 2 or 3 categories.[25, 28]

#### Assessment of covariates

Blood samples were collected by venipuncture after 10 to 12 hours of fasting, and the Seoul Medical Science Institute (SMSI), a laboratory certified by the Korean Ministry of Health and Welfare, carried out all blood analyses. Demographic and socioeconomic information was obtained from a health interview. For the body weight status evaluation, objective weight status was determined according to a respondent’s body mass index (BMI; weight in kilograms divided by the square of the height in meters), which was based on anthropometric measurements from the Health Examination Survey. Information regarding recent weight changes was also collected. The subjects were divided into three groups based on the body weight changes over the past one year: weight gain (increase more than 3 kg), no weight change (increase or decrease equal to or less than 3 kg), and weight loss (loss more than 3 kg).

IOP was measured with a Goldmann applanation tonometer. For subjects with unilateral glaucoma, IOP from the glaucomatous eye was chosen for analyses. For subjects without glaucoma or bilateral glaucoma, one eye was randomly chosen for IOP analyses. Systolic blood pressure (SBP) and diastolic blood pressure (DBP) for each participant was measured three times at 5-min intervals using a standard mercury sphygmomanometer (Baumanometer, WA Baum Co. Inc., Copiague, NY, USA) with the subject in a sitting position. The average of the second and third measurements was recorded as the final blood pressure measurement. Mean arterial pressure was calculated as the sum of 2/3 diastolic blood pressure and 1/3 systolic blood pressure. Smoking status was determined using self-reporting with subjects classified as current smokers, past smokers or non-smokers. Alcohol consumption was assessed by subjects’ drinking behavior during the month prior to the interview.

### Statistical analysis

Complex sample analysis was used for the KNHANES data for weighting all values following statistical guidance from the Korea Centers for Disease Control and Prevention. The regression model was constructed following identification of potential confounding variables. All risk factors that were identified as being associated with a glaucoma diagnosis by univariate analysis with P value <0.1 as the cutoff point were then considered to be included in the multivariate analysis to assess the possible independent association between exercise-related parameters and glaucoma. Following ascertainment of such a possible association, odds ratios (OR) and 95% confidence intervals (CI) were estimated for each possible association. All statistical tests were two-sided with 95% CI, using SPSS statistical software (version 21.0; IBM Corp., Armonk, NY, USA).

## Results

### Population characteristics

Of the 11,246 subjects included in our analysis, 336 met the ISGEO criteria for glaucoma diagnosis in either eye (ISGEO category 1 = 186 eyes, ISGEO category 2 = 217 eyes, ISGEO category 3 = 5 eyes; 72 subjects had bilateral glaucoma), representing 2.7% (Standard Error: 0.2%) of the population sample with requisite information. **Table 1** presents information on demographic characteristics, comorbidities, and health-related behavior between those with and without glaucoma diagnosed using ISGEO criteria. Participants with glaucoma were older, more likely to be male than female, and with higher blood urea nitrogen (BUN), creatinine, mean arterial pressure, lower BMI and higher IOP than those without glaucoma. There was a higher percentage of subjects with weight loss over the past year in the glaucoma group versus those without the disease (21.9 [2.7] % vs. 12.9 [0.4] %, respectively).

**Table 1:** Demographic and general health characteristics of participants with and without glaucoma.

Characteristic^a^	Glaucoma (n = 336/11246, 2.7%)	No Glaucoma (n = 10910/11246, 97.3%)	P values
Demographics			
Age, mean (SE), y	58.07 (0.80)	53.76 (0.17)	<0.001
Women	174 (44.7%)	6313 (53%)	0.015
Health-related behavior			
Smoking status			
Non-smoker	194 (53.6%)	6556 (55.9%)	0.271
Past smoker	47 (13.7%)	1222 (10.7%)	
Current Smokers	95 (32.6%)	3132 (33.4%)	
Alcohol			
Never had alcohol in past one year	56 (18.8%)	1551 (15.6%)	0.682
Ever had any alcohol in past one year	211 (81.2%)	7222 (84.4%)	
Weight changes over the past year			
No change (< 3kg change)	225 (65.9%)	7677 (71.2%)	<0.001
Weight loss	70 (21.9%)	1464 (12.9%)	
Weight gain	33 (12.2%)	1571 (15.9%)	
Medical comorbidities			
Diabetic mellitus	49 (16.0%)	1098 (10.3%)	0.029
Hypertension	117 (31.5%)	2885 (22.8%)	0.003
Hyperlipidemia	39 (10.5%)	1256 (10.2%)	0.869
Serum Markers, mean (SE)			
BUN, mg/dL	15.59 (0.28)	14.8 (0.6)	0.002
Creatinine, mg/dL	0.87 (0.02)	0.83 (0.002)	0.014
Blood sugar, mg/dL	100.21 (1.67)	99.46 (0.25)	0.629
Cholesterol, mg/dL	194.56 (2.72)	193.31 (0.47)	0.644
Triglycerol, mg/dL	145.86 (6.55)	147.47 (1.65)	0.815
BMI^b^, mean (SE), kg/m^2^	23.61 (0.22)	24.01 (0.04)	0.074
Mean arterial pressure, mean (SE), mmHg	94.97 (0.84)	92.60 (0.19)	0.003
IOP, mean (SE), mmHg	15.43 (0.29)	14.02 (0.05)	<0.001
Spherical equivalent, mean (SE), diopter	-0.70 (0.18)	-0.53 (0.03)	0.317

Abbreviations: BUN, blood urea nitrogen; BMI, body mass index; IOP, intraocular pressure.

^a^ Data are presented as number (percentage) of study participants unless otherwise indicated. Percentages may not total 100 because of missing values in the database and because percentages are weighted based on sampling methods used in the KNHANES

^b^ Calculated as weight in kilograms divided by height in meters squared.

### Physical activity patterns and glaucoma

There were more glaucoma subjects ascertained to be undertaking vigorous and moderate exercise 7 days per week, as well as walking 0 day per week compared with non-glaucomatous controls (6.5 [2.0] % vs. 3.0 [0.2] % for vigorous exercise 7 days per week; 10.9 [2.5] % vs. 7.0 [0.4] % for moderate exercise 7 days per week; 22.2 [2.8] % vs. 17.8 [0.5] for walking 0 day per week). Glaucoma subjects tended to have higher total minutes per week of doing moderate intensity exercise (**Table 2**).

**Table 2:** Patterns of physical activity for patients with and without glaucoma.

Patterns of physical activity (# in each category)	Glaucoma (n = 336)	No Glaucoma (n = 10910)
	Fraction, weighted %, (SE)	Fraction, weighted %, (SE)
Q1. How many days did you perform at least 10 minutes vigorous physical activity?		
0 day/week (7771)	239/336 = 66.1 (3.4)	7532/10910 = 65.4 (0.7)
1 day/week (946)	20/336 = 7.9 (2.0)	926/10910 = 9.6 (0.4)
2 days/week (753)	25/336 = 8.7 (2.0)	728/10910 = 7.7 (0.3)
3 days/week (700)	18/336 = 6.0 (1.6)	682/10910 = 6.8 (0.3)
4 days/week (268)	3/336 = 0.9 (0.6)	265/10910 = 2.9 (0.2)
5 days/week (322)	8/336 = 2.0 (0.9)	314/10910 = 3.0 (0.2)
6 days/week (159)	6/336 = 1.9 (0.9)	153/10910 = 1.6 (0.2)
7 days/week (327)	17/336 = 6.5 (2.0)	310/10910 = 3.0 (0.2)
Vigorous exercise, total minutes/week, mean (SE)	116.0 (18.1)	126.0 (4.9)
Q2. How many days did you perform at least 10 minutes moderate physical activity?		
0 day/week (6664)	202/336 = 56.1 (3.4)	6462/10910 = 57.9 (0.8)
1 day/week (874)	20/336 = 6.7 (1.9)	854/10910 = 8.4 (0.3)
2 days/week (918)	24/336 = 6.5 (1.5)	894/10910 = 8.7 (0.3)
3 days/week (810)	28/336 = 11.1 (2.4)	782/10910 = 7.4 (0.3)
4 days/week (407)	11/336 = 2.1 (0.7)	396/10910 = 3.9 (0.2)
5 days/week (471)	15/336 = 5.3 (1.6)	456/10910 = 4.2 (0.3)
6 days/week (261)	6/336 = 1.3 (0.6)	255/10910 = 2.5 (0.2)
7 days/week (841)	30/336 = 10.9 (2.5)	811/10910 = 7.0 (0.4)
Moderate exercise, total minutes/week, mean (SE)	224.7 (30.2)	195.3 (8.1)
Q3. How many days did you perform at least 10 min. walking?		
0 day/week (2064)	82/336 = 22.2 (2.8)	1982/10910 = 17.8 (0.5)
1 day/week (690)	17/336 = 5.8 (1.7)	673/10910 = 6.3 (0.3)
2 days/week (1063)	23/336 = 9.4 (2.1)	1040/10910 = 10.2 (0.4)
3 days/week (1454)	49/336 = 14.7 (2.4)	1405/10910 = 13.2 (0.4)
4 days/week (756)	17/336 = 4.4 (1.3)	739/10910 = 7.0 (0.3)
5 days/week (971)	29/336 = 7.0 (1.4)	942/10910 = 8.7 (0.3)
6 days/week (571)	13/336 = 4.3 (1.5)	558/10910 = 5.5 (0.3)
7 days/week (3677)	106/336 = 32.2 (3.5)	3571/10910 = 31.3 (0.6)
Walking, total minutes/week, mean (SE)	313.8 (32.8)	348.0 (7.8)
Q4. How many days did you do exercise for muscle strength?		
0 day/week (8228)	254/327 = 74.7 (3.1)	7974/10716 = 71.8 (0.7)
1 day/week (584)	13/327 = 5.3 (1.6)	571/10716 = 6.1 (0.3)
2 days/week (632)	17/327 = 4.3 (1.3)	615/10716 = 6.8 (0.3)
3 days/week (580)	16/327 = 5.2 (1.5)	564/10716 = 5.5 (0.3)
4 days/week (319)	9/327 = 4.1 (1.5)	310/10716 = 3.2 (0.2)
≥ 5 days/week (700)	18/327 = 6.4 (1.9)	682/10716 = 6.6 (0.3)
Q5. How many days did you do exercise for flexibility?		
0 day/week (5336)	168/327 = 49.0 (3.5)	5168/10717 = 45.8 (0.7)
1 day/week (852)	26/327 = 8.7 (2.0)	826/10717 = 8.6 (0.3)
2 days/week (1105)	32/327 = 11.0 (2.3)	1073/10717 = 11.0 (0.4)
3 days/week (1211)	34/327 = 9.5 (1.9)	1177/10717 = 11.1 (0.4)
4 days/week (609)	12/327 = 4.6 (1.5)	597/10717 = 6.0 (0.3)
≥ 5 days/week (1931)	55/327 = 17.3 (2.5)	1876/10717 = 17.5 (0.5)
Q6. I met vigorous physical activity guidelines^1^	52/326 = 17.9 (2.7)	1682/10713 = 17.2 (0.5)
Q7. I met moderate physical activity guidelines^2^	50/326 = 17.8 (2.9)	1359/10710 = 12.5 (0.5)
Q8. I met low physical activity guidelines^3^	128/336 = 38.5 (3.6)	4396/10910 = 39.9 (0.7)
Physical activity intensity (based on Q6-8)		
Low (not meet any of Q6, Q7 and Q8)	175/336 = 52.3 (3.5)	5377/10910 = 49.3 (0.7)
Moderate (meet 1/3 of Q6-Q8)	109/336 = 29.3 (3.0)	3972/10910 = 35.9 (0.6)
High (meet ≥ 2/3 of Q6-Q8)	52/336 = 18.4 (2.9)	1561/10910 = 14.8 (0.5)

^1^ 20 minutes vigorous physical activity ≥ 3 times/week

^2^ 30 minutes moderate physical activity ≥ 5 times/week

^3^ 30 minutes walking ≥ 5 times/week

**Table 3** demonstrates demographic characteristics, comorbidities, and health-related behavior among subjects with three different physical activity levels. Subjects with high physical activity intensity were more likely to be male. There were more subjects with weight loss during the preceding year but also less people with BMI less than 19 kg/m^2^ in the high intensity physical activity group while compared with the other groups. There were more subjects with weight gain more than 3 kg in the moderate intensity group compared with the other two groups. While there were more current smokers in the high intensity group than moderate and low intensity groups, the prevalence of medical comorbidities such as diabetic mellitus, hypertension, and hyperlipidemia were not higher in the high intensity exercise group. There was no difference among groups regarding mean arterial pressure and IOP.

**Table 3:** Demographic and general health characteristics of participants in low, moderate and high physical activity intensities.

Characteristic^a^	Low (n = 5552, 49.4%)	Moderate (n = 4081, 35.7%)	High (n = 1613, 14.9%)
Demographics			
Age, mean (SE), y	53.8(0.2)	54.5 (0.2)	52.47 (0.33)
Women (weighted %)	3326 (54.3)	2295 (52.3)	866 (48.8)
Health-related behavior (weighted %)			
Smoking status			
Non-smoker	3460 (58.2)	2370 (53.8)	920 (53.0)
Past smoker	605 (10.4)	493 (11.4)	171 (10.2)
Current Smokers	1487 (31.4)	1218 (34.8)	522 (36.9)
Weight changes over the past year (weighted %)			
No change (< 3kg change)	3859 (72.1)	2940 (62.1)	1103 (67.9)
Weight loss	684 (11.9)	571 (15.1)	279 (16.2)
Weight gain	804 (16.0)	569 (22.8)	231 (15.9)
Medical comorbidities (weighted %)			
Diabetic mellitus	552 (10.6)	430 (10.5)	165 (9.7)
Hypertension	1497 (23.0)	1119 (23.9)	386 (20.8)
Hyperlipidemia	630 (10.3)	499 (10.6)	166 (9.2)
Serum Markers, mean (SE), mg/dl			
BUN	14.7 (0.1)	14.85 (0.09)	15.07 (0.14)
Creatinine	0.83 (0.003)	0.84 (0.005)	0.83 (0.006)
Blood sugar	99.6 (0.4)	99.37 (0.43)	99.3 (0.67)
Cholesterol	193.6 (0.6)	193.80 (0.75)	191.6 (1.27)
Triglycerol	151.9 (2.4)	146.21 (2.70)	136.02 (3.98)
BMI^b^, mean (SE), kg/m^2^	23.98 (0.05)	23.97 (0.06)	24.15 (0.1)
<19 kg/m^2^	273 (4.6)	154 (3.7)	53 (3.1)
19–24.9 kg/m^2^	3292 (59.6)	2501 (61.2)	950 (59.1)
≥25 kg/m^2^	1962 (35.8)	1413 (35.1)	606 (37.8)
Mean arterial pressure, mean (SE), mmHg	92.33 (0.24)	93.0 (0.3)	92.98(0.42)
IOP, mean (SE), mmHg	14.04 (0.06)	14.1 (0.1)	14.08 (0.1)
Spherical equivalent, mean (SE), diopter	-0.60 (0.04)	-0.47 (0.04)	-0.49 (0.07)

Abbreviations: BUN, blood urea nitrogen; BMI, body mass index; IOP, intraocular pressure.

^a^ Data are presented as number (percentage) of study participants unless otherwise indicated. Percentages may not total 100 because of missing values in the database.

^b^ Calculated as weight in kilograms divided by height in meters squared.

In a multivariable analysis model using moderate physical activity subjects as comparators, high intensity (OR = 1.55; 95% CI: 1.03–2.33) but not low intensity exercise (OR = 1.31; 95% CI: 0.95–1.81) was associated with greater glaucoma prevalence (**Table 4**). Furthermore, after adjusting for confounders, greatest frequency of performing vigorous exercise (7 days per week compared with 3 days per week as recommended by ACSM) was associated with higher odds of a glaucoma diagnosis (OR 3.33, 95% CI 1.16–9.54; **Table 4**). In a secondary analysis, compared to moderate physical activity level, both low intensity physical activity (OR = 1.71; 95% CI: 1.09–2.69) and high intensity physical activity (OR = 2.19; 95% CI: 1.25–3.84) were adversely associated with glaucoma in men, but not in women (**Table 5**). In women there was no association between intensity of physical activity and glaucoma prevalence (**Table 5**). Among men, compared to engaging in vigorous exercise for at least 10 minutes 3 days per week, engaging in vigorous exercise 7 days per week was associated with increased risk of glaucoma (OR = 6.05; 95% CI: 1.67–21.94) (**Table 6**). Among women, there was no association between vigorous exercise frequency and glaucoma (**Table 6**).

**Table 4:** Odds ratios (OR) and 95% confidence intervals (CI) for the presence of glaucoma among different levels of physical activity and frequency of vigorous exercise.

	Un-adjusted OR (95% CI)	Adjusted OR (95% CI)
Physical activity intensity ^b^		
Low	1.30 (0.96–1.75)	1.31 (0.95–1.81) ^a^
**Moderate**	**1.00 (reference)**	**1.00 (reference)** ^a^
High	1.52 (1.01–2.30)	1.55 (1.03–2.33) ^a^
Vigorous exercise, days/week, % (SE)^c^		
0 day/week	1.16 (0.66–2.03)	0.77 (0.40–1.49) ^d^
1 day/week	0.95 (0.45–2.00)	0.83 (0.38–1.81) ^d^
2 days/week	1.29 (0.66–2.53)	1.35 (0.68–2.69) ^d^
**3 days/week**	**1.00 (reference)**	**1.00 (reference)** ^d^
4 days/week	0.36 (0.091–1.44)	0.43 (0.11–1.73) ^d^
5 days/week	0.74 (0.26–2.15)	0.82 (0.28–2.42) ^d^
6 days/week	1.36 (0.44–4.19)	1.91 (0.63–5.78) ^d^
7 days/week	2.50 (1.03–6.04)	3.33 (1.16–9.54) ^d^
Vigorous exercise, total minutes/week, mean (SE)	0.99 (0.99–1.00)	0.999 (0.999–1.00) ^d^

^a^ Multivariable analysis adjusted for age, gender, IOP, spherical equivalent, mean arterial pressure, weight changes over the past year, BMI, and history of diabetes.

^b^ Subjects were questioned regarding the level of physical activity in the following 3 categories: (a) Did you walk for at least 30 minutes, 5 times on a recent week? (b) Did you perform moderate physical activity for at least 30 min, 5 times on a recent week? and (c) Did you perform vigorous physical activity for at least 20 minutes, 3 times on a recent week? The physical activity intensity was divided in 3 different levels: (a) low, subjects who did not belong in any of 3 categories; (b) moderate, subjects who belong to 1 of the 3 categories; and (c) high, subjects who belong to 2 or 3 categories.

^c^ Vigorous physical activities include running or jogging, hiking, fast bike riding, fast swimming, football, basketball, jumping rope, squash, singles tennis, heavy items lifting and athletic activities, for at least 10 minutes a day.

^d^ Multivariable analysis adjusted for age, gender, IOP, spherical equivalent, mean arterial pressure, total minutes/week of vigorous exercise, weight changes over the past year, BMI, and history of diabetes

**Table 5:** Association between intensity of physical activity and glaucoma in men and women.

		Glaucoma, % (SE)	Adjusted OR (95% CI)^a^
	Physical activity intensity ^b^		
Men	Low	3.5 (0.5)	1.71 (1.09–2.69)
	**Moderate**	**2.2 (0.4)**	**1.00 (reference)**
	High	4.1 (0.9)	2.19 (1.25–3.84)
Women	Low	2.3 (0.3)	0.95 (0.61–1.47)
	**Moderate**	**2.2 (0.4)**	**1.00 (reference)**
	High	2.5 (0.7)	1.02 (0.56–1.85)

^a^ Multivariable logistic model adjusted for age, gender, IOP, spherical equivalent, mean arterial pressure, weight changes over the past year, BMI, history of diabetes

^b^ Subjects were questioned the level of physical activity in the following 3 categories: (a) Did you walk for at least 30 min, 5 times on a recent week? (b) Did you perform moderate physical activity for at least 30 min, 5 times on a recent week? and (c) Did you perform vigorous physical activity for at least 20min, 3 times on a recent week?

The physical activity intensity was divided in 3 different levels: (a) low, subjects who did not belong in any of 3 categories; (b) moderate, subjects who belong to 1 of the 3 categories; and (c) high, subjects who belong to 2 or 3 categories.

**Table 6:** Association between frequency of doing vigorous exercise and glaucoma in men and women.

Gender		Glaucoma, % (SE)	No glaucoma, % (SE)	Adjusted OR (95% CI)^a^
Men (7741, 49.1%)		N = 162, 3.2 (0.3)	N = 4597, 96.8 (0.3)	
	Vigorous exercise			
	0 day/week	58.0 (4.7)	57.6 (0.9)	0.97 (0.44–2.17)
	1 day/week	8.4 (2.6)	12.8 (0.6)	0.80 (0.30–2.14)
	2 days/week	12.4 (3.2)	9.6 (0.5)	1.82 (0.76–4.32)
	**3 days/week**	**5.5 (1.9)**	**7.8 (0.5)**	**1.00 (reference)**
	4 days/week	0.3 (0.3)	3.4 (0.3)	0.13 (0.02–1.06)
	5 days/week	3.2 (1.6)	3.4 (0.3)	1.49 (0.43–5.1)
	6 days/week	2.6(1.6)	2.3 (0.3)	2.38 (0.60–9.5)
	7 days/week	9.6 (3.3)	3.1 (0.3)	6.05 (1.67–21.94)
	Total minutes per week, mean (SE)	149.88 (28.17)	157.45 (8.10)	0.999 (0.999–1.00)
Women (10645, 50.9%)		N = 174, 2.3 (0.2)	N = 6313, 97.7 (0.2)	
	Vigorous exercise			
	0 day/week	76.1 (4.2)	72.3 (0.8)	0.59 (0.19–1.78)
	1 day/week	7.4 (3.0)	6.7 (0.4)	1.03 (0.29–3.70)
	2 days/week	4.1 (1.6)	6.1 (0.4)	0.68 (0.19–2.39)
	***3* days/week**	**6.5 (2.7)**	**6.0 (0.4)**	**1.00 (reference)**
	4 days/week	1.7 (1.2)	2.4 (0.3)	0.73 (0.22–3.84)
	5 days/week	0.4 (0.4)	2.7 (0.3)	0.18 (0.02–1.66)
	6 days/week	1.0 (1.7)	1.0 (0.1)	1.44 (0.23–8.88)
	7 days/week	2.8 (1.2)	2.9 (0.3)	0.96 (0.23–3.97)
	Total minutes per week, mean (SE)	74.04 (17.19)	98.02 (4.91)	0.999 (0.998–1.00)

^a^ Multivariable logistic model adjusted for age, gender, IOP, spherical equivalent, mean arterial pressure, total minutes/week of vigorous exercise, weight changes over the past year, BMI, history of diabetes

We did not find similar associations between exercise duration and glaucoma, between frequency of doing moderate exercise or walking or between muscle strength or flexibility training, with glaucoma (all P >0.05; data not shown).

## Discussion

We investigated the relationship between exercise and glaucoma risk in the South Korean population, and after adjustment for potential confounding variables including BMI, IOP and diabetes our results revealed a strong positive association between the frequency of performing vigorous exercise and glaucoma prevalence. Male subjects demonstrated both low and high levels of exercise intensity to be associated with greater glaucoma prevalence compared to moderate intensity exercise as recommended in the ACSM guidelines.

Exercise has generally been considered to be beneficial in terms of ocular health with one report showing an associated reduction in central retinal vein occlusion by half.[1] Other work has shown an exercise-related reduction in neovascular age-related macular degeneration by as much as 70%[2], and improved control of systemic hypertension as well as diabetes.[4] Exercise also increases ocular perfusion pressure by increasing systolic blood pressure and reducing IOP in the short-term.[5, 6] Chrysostomou et al. demonstrated that daily swimming in middle-aged mice protected retinal ganglion cells (RGCs) against age-related functional loss and signs of stress after an acute injury while compared with no exercise controls.[3] This protective effect was found to be associated with levels of brain-derived neurotrophic factor (BDNF) in the retina, with the resultant generation of a hypothesis that exercise promotes improves neuronal health in the retina by maintaining adequate levels of BDNF in injured RGCs thereby preventing complement-mediated synapse elimination.[29]

Despite the theoretical benefits of exercise based upon preclinical experiments, we found higher glaucoma prevalence in subjects who participated in high versus moderate intensity physical activity. In addition, daily vigorous exercise was associated with a higher prevalence of glaucoma as compared with similar intensity exercise performed 3 days per week as recommended by ACSM. A recent study has shown that free radicals accumulated from exhaustive exercise causes structural damage or inflammatory reactions within muscle tissue[15]. An increasing body of evidence also suggests that oxidative stress plays a major role in glaucomatous disease[16, 17], and a high level of lipid peroxidation products and mitochondrial DNA damage are found in tissues from the trabecular meshwork of patients with primary open angle glaucoma, with the possible consequence being an increase in IOP.[16, 18, 30] Such oxidative stress may also be harmful to retinal ganglion cells, with resultant cell death leading to optic nerve damage characteristic of glaucomatous disease.[31] The premise that aerobic exercise lowers IOP in the short-term is widely accepted in the literature[4, 32], although the mechanism is poorly understood.[5] However, certain physical activities such as weightlifting and yoga can cause transient IOP spikes by increasing intra-thoracic and abdominal pressure. In turn these effects can increase episcleral venous pressure and reduce aqueous outflow, which may be transmitted to the choroid, causing choroidal expansion[33, 34], and ultimately increased IOP.[35] Dickerman described a sudden large rise in IOP following Valsalva maneuver in power athletes, which in some subjects reached 46 mmHg. Mean IOP rose from 13 mmHg at rest to 28 mmHg during isometric contraction, and these large pressure changes lasted for 3 to 8 seconds.[12, 13] In the present study, we found a positive relationship between the frequency of performing vigorous exercise and glaucoma prevalence, but could not distinguish the relative impact of different types of exercise, which warrants further investigation.

Generally, ocular blood perfusion is maintained constantly and adequately by autoregulation during the perfusion pressure changes from the effect of exercise.[7–9] Retinal[36], choroidal[8, 37], and optic nerve head blood flow[38, 39] are regulated to ideally remain constant during exercise. At high levels of exertion OPP can increase by 40–60% at which point limits of regulation are exceeded and blood flow has been demonstrated to increase in a linear manner.[4] This generally protective mechanism maybe harmful in some patients such as those with diabetic autonomic neuropathy who show less retinal vasoconstriction and regulation of blood flow during exercise compared to patients without neuropathy or diabetes.[10, 11]

Multiple lines of evidence also exist for vascular dysregulation in patients with open-angle glaucoma. Dysregulated blood flow has been described in the choroid[40, 41], the optic nerve[42, 43], the central retinal artery[44, 45] as well as the perifoveal macular capillaries.[46] Feke and Pasquale showed that simple positional change[47] could result in dramatic changes in retinal arteriole blood flow and suggested that such changes could trigger disc hemorrhages, which were biomarkers for glaucoma progression in the Ocular Hypertension Treatment Study[48], the Early Manifest Glaucoma Trial[49], and the Collaborative Normal Tension Glaucoma Study[50]. It is possible that excessive exercise could cause even more dramatic retinal hemodynamic changes leading to optic nerve ischemic reperfusion injury, regardless of the prevailing IOP in OAG patients. Autonomic dysfunction with low beat-to-beat cardiac variability[51, 52] and relatively monotonous cardiac power[53] could exacerbate this ischemia-reperfusion injury in the face of extreme exercise. However, there is no direct evidence that exercise has either beneficial or harmful effects on ocular blood flow in the medium or long term. In a population with a high prevalence of normal tension glaucoma such as in South Korea[54], vascular disease etiology may be implicated as contributing to this chronic disease process.[55–57] Therefore, advances in non-invasive techniques for measuring the effects of exercise on ocular blood flow are needed to provide information on wider aspects of vascular physiologic changes associated with exercise and its impact on eye health.[58]

The gender specific findings noted in our study were surprising. Amongst male subjects, we found that both the low and high intensity exercise groups, as defined by the guidelines of ACSM, had higher glaucoma prevalence compared with the moderate intensity exercise group. This relationship was not found in women. It could be that high physical activity overwhelms a dysregulated cardiovascular system and subsequently leads to glaucoma.[51–53] One possible reason for only the extreme level of high physical activity intensity (exercising vigorously 7 days a week) showing a relation to glaucoma when both sexes are considered could be because of the higher ratio of men in the high intensity group and more women in the low intensity group in our study population with gender serving as a confounding variable (Table 3). Future prospective, longitudinal studies are needed to clarify the relationship between exercise pattern and glaucoma in men versus women.

Our study has several limitations. First, we were not able to calculate the total activity scores (calculated by multiplying the intensity of the specific types of activities, defined as metabolic equivalents [MET; kilocalories per kilograms per hour], by the duration)[59] or measure relevant biomarker levels such as the lactate threshold[60], which could quantify the exercise intensity more precisely. There are several forms of wearable technology to record physical activity, which could perhaps be employed in future studies to address these shortcomings. Although we were not able to investigate how the self-reported activity levels correlate with objectively measured activity levels in KNHANES, we do see reasonable construct validity for our self reported exercise measures and other covariates such as greater weight loss, less hypertension and lower serum cholesterol and triglycerol in the high intensity exercise group (Table 3). Random misclassification of self-reported exercise activity would have driven the results to the null but we found robust adverse association between vigorous exercise and glaucoma, especially among men. It is noteworthy that we could not differentiate the types of exercise and it is likely that some physical activities such as weightlifting and yoga can cause transient IOP elevation, possibly contributing to glaucomatous damage in vulnerable populations.[12, 61] Further, intense aerobic vs anerobic exercise may have very different effects on glaucoma risk. We did not assess the relationship between IOP and exercise since there is no information regarding IOP immediately after exercise in KNHANES, and there is no confirmatory evidence that physical activity has an effect on resting IOP in the long-term.[4] In addition, it must be acknowledged that our work simply demonstrated an association between the frequency of performing vigorous exercise and glaucoma prevalence without shedding light on causality which cannot be adequately assessed in such cross sectional studies.

Previous studies demonstrated the impact of glaucomatous VF loss on physical activity performed during the normal lives of patients. Ramulu et al. found no overall decrease in physical activity in glaucoma subjects, though secondary analyses demonstrated that physical activity levels were lower with greater levels of VF loss.[62] Landingham et al. showed individuals with bilateral VF loss took 17% fewer steps per day and engaged in 30% less daily minutes of moderate or vigorous physical activity than individuals without VF loss.[63] We did not evaluate the relationship of glaucoma severity or the degree of VF loss versus the level of physical activity in our study population. Furthermore, there was only one FDT test obtained for each participant in KNHANES. The rigorous 2-2-1 rule for VF interpretation suggested by previous investigators was not employed.[64] In an effort to minimize false positive diagnoses, we excluded participants with retinal disease or a history of stroke thereby likely reducing error related to inclusion of subjects who may have had VF defects due to non-glaucomatous conditions.

In summary, after adjusting for potential confounders, we found that the performance of high intensity and vigorous exercise 7 days per week was associated with a greater likelihood of a glaucoma diagnosis in a representative sample of the South Korean adult population. In contrast, the total time of vigorous exercise was not associated with glaucoma. Future large prospective investigations are warranted to confirm or refute these findings, and to better understand the exercise-related vascular responses of the eye as well asthe body’s adaption or exhaustion from oxidative stress related to the performance of vigorous exercise. Such studies would be necessary prior to making definitive recommendations regarding ideal exercise patterns for individuals at risk for glaucomatous disease.
